# Intrinsic drive of medical staff: a survey of employee representatives from 22 hospitals in China

**DOI:** 10.3389/fpsyg.2023.1157823

**Published:** 2023-04-27

**Authors:** Yuqing Zhang, Zheng Yuan, Taozhu Cheng, Cunliang Wang, Jun Li

**Affiliations:** ^1^Department of Health Administration and Policy, School of Public Health, Capital Medical University, Beijing, China; ^2^Department of Education, Beijing Hospital of Traditional Chinese Medicine, Beijing, China; ^3^Department of Health Policy and Management, School of Public Health, Peking University, Beijing, China; ^4^Department of Labour Union, Beijing Hospitals Authority, Beijing, China

**Keywords:** intrinsic drive, medical staff, employee representatives, motivation, turnover intention

## Abstract

**Objective:**

While several initiatives, including monetary rewards and performance system reform, are used to inspire medical staff, none are fully effective. We sought to describe the intrinsic drive of medical staff and identify elements that improve work enthusiasm by increasing internal motivation.

**Methods:**

A cross-sectional study was conducted in which 2,975 employee representatives from 22 municipal hospitals in Beijing, China were interviewed using a self-made intrinsic motivation scale for medical staff which includes the achievement motivation, self-efficacy, conscientiousness, gratitude level and perceived organizational support. The Kruskal-Wallis analysis of variance and multiple linear regression methods were used to investigate the level of intrinsic motivation and identify any influencing factors. The correlation between employee drive and turnover intention was determined using Spearman rank correlation analysis and Kendall’s tau b rank correlation coefficient.

**Results:**

A total of 2,293 valid answers were obtained, with a valid recovery rate of 77.1%. There were statistically significant differences in intrinsic motivation and its five dimensions by marital status, political status, profession, service year, monthly income, number of working hours per week, and turnover intention (*p* < 0.05). Being divorced, a CPC member, in the nursing profession, and having a higher monthly income had a positive impact on intrinsic motivation while working a high hours per week had a negative effect. Higher work drive was associated with lower turnover intention. The correlation coefficients of intrinsic drive and its five dimensions with turnover intention ranged from 0.265 to 0.522 (*p* < 0.001).

**Conclusion:**

Sociodemographic factors and work environment influenced the intrinsic motivation of medical staff. There was a correlation between work drive and turnover intention which indicated that stimulating the intrinsic drive of employees may help to increase staff retention.

## 1. Introduction

Motivating and improving the behavior of medical staff has been a key issue in China since healthcare reform was initiated in 2009, and has been explored by many other countries worldwide. However, extrinsic incentives such as monetary rewards, performance system reform, and criminalization of injury to doctors, are not fully effective (Alrawahi et al., 2020; Hartzband and Groopman, 2020). The United States has used physician performance incentives for the past decade, gaining significant attention through the Affordable Care Act (Cassel and Jain, 2012). However, one study found that such incentives do not improve and may even hinder healthcare services (Khullar et al., 2018) by inhibiting intrinsic motivation and reducing performance (Deci et al., 1999). Recent findings illustrate that green intrinsic motivation can improve employee creativity and green extrinsic motivation can reduce intrinsic motivation to a certain extent (Li et al., 2020). Some studies also show that incentives and intrinsic motivation are not necessarily antagonistic and may be considered simultaneously, and intrinsic motivation remains critical to performance regardless of whether incentives are given (Cerasoli et al., 2014).

According to social psychologists, individuals with intrinsic motivation often have a strong sense of work accomplishment, self-efficacy, and job satisfaction. Intrinsic motivation helps to increase learning, boost efficiency, and encourage creativity (Burton et al., 2006). Autonomous motivation, which is self-determined, is associated with lower burnout, higher job satisfaction, and a lower propensity to leave (Moller et al., 2019). Some researchers have considered influencing the behavior of medical employees by rationally matching their external and internal motivation (Deci and Ryan, 1980; Judson et al., 2015; Kao, 2015) and stimulating resilience by promoting the self-regulation of stress (Anandarajah et al., 2018).

Internal factors have a significant impact on employee motivation. Thus, it is critical to be aware of the work status of medical personnel to ensure that they remain motivated to provide high-quality clinical services, especially given the recent increase in work-related burnout (Greep et al., 2021; Tiete et al., 2021; Lou et al., 2022) and the rise in anxiety and depression associated with the COVID-19 pandemic (Elbay et al., 2020; Gainer et al., 2021; Tuna and Zdin, 2021). The comprehension of intrinsic motivation has been viewed in several different ways. In the early 20th century Woodworth suggested that intrinsic motivation governs activities perpetuated by an individual’s “native drive” (Woodworth, 1918). Jung had the view that drives are biological signals that have a driving effect and give positive cues to an individual (Jung, 1966) while Ausubel proposed that achievement motivation consists of cognitive, self-improvement, and subsidiary internal drives (Ausubel, 1968). It has also been suggested that drive is triggered by external stimulations and that medical students are intrinsically motivated by their roles, clinical experience, patient interactions, and peer communication (Kim et al., 2022). The subjective nature of intrinsic motivation has made it difficult to quantify. Some scholars have drawn on the “exploration-exploit” paradigm to measure motivation (Robbins, 1952; Gittins and Jones, 1979; Morris et al., 2022). There are also scales that measure intrinsic motivation for reading from the interest-enjoyment dimension (Woolley and Fishbach, 2018), and instruments to measure physician motivation that include intrinsic and extrinsic influences (Malik et al., 2018). However, no systematic and targeted studies have been conducted that integrate positive intrinsic work motivation among medical staff, and no suitable tool has been developed to evaluate this factor.

We compared the concepts of “drive” in psychology, “internal cause” in philosophy, and “internal motivation” in management, in combination with the characteristics of saving lives and putting life first, and defined the “intrinsic drive”(ID) of medical staff as “the internal motivation of medical staff that is internalized as an autonomous and spontaneous drive in the process of socialization based on the general perceptions of the growing environment, without the pursuit of reward.” Researches have shown that achievement motivation, self-efficacy, conscientiousness and gratitude level are important components of ID, respectively. Perceived organizational support is also closely related to ID, which has a direct and strong impact on it. A model of ID was constructed with five dimensions: achievement motivation (AM), self-efficacy (SE), conscientiousness (CS), gratitude level (GL), and perceived organizational support(POS) (Zhang et al., 2021). To further quantify ID for research purposes, relevant measurement scales were developed (Zhang and Li, 2021). First, a pool of entries was established through theoretical models and psychological scales, and the entries were refined through Delphi expert consultation. Second, three surveys (64, 891, and 1,435 participants) were conducted, and the scale was revised after each questionnaire using common method biases tests, discrimination analysis, reliability analysis, validity analysis, and model fit tests, and finally resulting in the Medical Staff Internal Drive Scale (IDS) (the flowchart for building the scale is shown in Supplementary material).

This is the first study to analyze the intrinsic drive of medical staff. Future studies are needed to better understand possible intrinsic incentives for medical staff and ways to improve their practice techniques. One study used the ID scale (IDS) to survey ICU medical staff, and the Cronbach’s *α* coefficient was 0.953, indicating high reliability. A positive correlation was found between ID and professional identity, indicating that it would be worthwhile understanding this correlation to help motivate medical staff to work actively (*r* = 0.17 ~ 0.86, *p* < 0.05) (Feng et al., 2022). The current study used an IDS as a tool to study the intrinsic drive of employee representatives in 22 hospitals in Beijing. The findings provide a reference for enhancing employee drive and improving work enthusiasm.

## 2. Methods

### 2.1. Study design and participants

A descriptive cross-sectional survey study was conducted to assess the internal motivation level of medical staff. Large hospitals in Beijing are owned by various entities, including the central government, Beijing municipal government, businesses and so on, and hospital management under different ownership types can differ significantly. The Beijing municipal government has the highest number of large hospitals in its jurisdiction, and available data from these hospitals were sufficient to conduct the analysis. A total of 2,975 staff representatives from 22 municipal hospitals in Beijing from December 2020 to January 2021 were included in this study (the names of the 22 hospitals are shown in Supplementary material). Of these, 20 were grade A tertiary hospitals and two were tertiary hospitals. At the level of hospital type, 15 were general and seven were specialized. According to the Worker’s Congress of Beijing-affiliated hospitals in China, staff representatives should cover various positions in the hospital, including front-line staff, technicians, administrators, and leaders. There are also regulations on the proportion of positions filled by administrators, women, and younger individuals. Staff representatives are directly elected by hospital employees and have a certain degree of representativeness. Through “Questionnaire Star,” a platform for creating, disseminating, and collecting electronic questionnaires, the labor union of each hospital distributed the study survey to the employee representative, who voluntarily filled it out.

### 2.2. Questionnaire and data acquisition

The previously developed IDS for medical staff was used to assess each respondent’s ID. The scale includes nine questions on AM, four questions on SE, two questions on CS, two questions on GL, and 12 questions on POS. A total of 30 questions were scored on a 7-point Likert scale, of which eight were reverse scored. Covariates include demographic variables (gender, age, marital status, education, political status) and occupation-related variables (profession, service year, professional title, weekly working hours, monthly income, turnover intention).

### 2.3. Quality control

Quality control of the questionnaire was achieved through five steps. First, the questionnaire was made anonymous to guarantee that participants answered truthfully. Second, the dimensional questions were randomized to avoid systematic response errors. Finally, reverse questions were designed to prevent stereotyped thinking of the research subjects and allow invalid responses to be identified.

Once the questionnaires were collected, the invalid responses were screened by the response time. The shortest response time was calculated after multiple testing, based on careful completion of the questionnaires, and only the time spent filling out the questionnaire that exceeded this period was included in subsequent analysis (the time spent answering the questionnaire can be observed and exported from Questionnaire Star’s background). Second, the “double-question rule” was used to screen for invalid responses by assessing whether two questions with similar content but opposite expressions had the tendency to be answered the same. For example, if questions 1 and 3 were similar in content but opposite in expression, then the questionnaire was considered invalid if the respondents answered “somewhat agree,” “agree,” or “completely agree” OR “somewhat disagree,” “disagree,” or “completely disagree” for both questions.

### 2.4. Statistical analysis

Covariate data were summarized using descriptive statistics. The level of ID and its five dimensions were expressed using the mean ± standard deviation (*M* ± *SD*). Kruskal-Wallis (*K-W*) variance analysis was performed for the main indicators using IBM SPSS Statistics software version 25.0. Confirmatory factor analysis was performed using Amos software version 26.0. The multiple linear regression method was used to investigate the factors that influenced medical staff ID. Spearman rank correlation analysis and the Kendall’s tau_b rank correlation coefficient were used to assess the relationship between ID and turnover intention. The threshold for statistical significance was set at *p* < 0.05 and all tests were two-tailed.

## 3. Results

### 3.1. Sociodemographic factors and intrinsic drive

A total of 2,975 questionnaires and 2,293 valid answers were obtained after quality control, yielding an effective recovery rate of 77.1% (a rate > 70% is considered a valid reference for research conclusions) (Babbie, 2007). The results of this population may have distinct features since some respondents worked in multiple occupations, including as both physicians and administrators and these 74 persons were counted individually. The ID by each covariate is shown in Table 1. The results indicated that a quarter of respondents wanted to quit their jobs “sometimes,” “often,” or “always.”

**Table 1:** Demographic information, level of intrinsic drive and its five dimensions (*M ± SD*).

Characteristics	*N* (%)	AM	SE	CS	GL	POS	ID
Gender							
Male	675 (29.4)	54.69 ± 6.55	24.14 ± 3.15	12.43 ± 1.56	18.59 ± 2.22	63.56 ± 15.1	34.25 ± 4.76
Female	1,618 (70.6)	54.57 ± 6.5	23.88 ± 3.31	12.53 ± 1.52	18.64 ± 2.25	64.64 ± 14.24	34.4 ± 4.71
*p*		0.465	0.103	0.145	0.429	0.165	0.472
Age							
Under 30	213 (9.3)	53.66 ± 6.87	23.68 ± 3.33	12.35 ± 1.63	18.35 ± 2.44	63.48 ± 15.01	33.86 ± 5
31–40	725 (31.6)	54.14 ± 6.9	23.71 ± 3.43	12.37 ± 1.62	18.47 ± 2.39	63.98 ± 14.57	34.08 ± 4.99
41–50	763 (33.3)	54.67 ± 6.21	23.94 ± 3.24	12.49 ± 1.51	18.67 ± 2.17	64.25 ± 14.3	34.36 ± 4.6
Over 50	592 (25.8)	55.43 ± 6.17	24.39 ± 3.02	12.73 ± 1.37	18.86 ± 2.05	65.14 ± 14.5	34.86 ± 4.4
*p*		0.001**	0.002**	0.001**	0.043*	0.337	0.032*
Marital status							
Single	212 (9.2)	52.8 ± 6.84	23.16 ± 3.45	12.12 ± 1.69	18.24 ± 2.49	63.15 ± 14.13	33.44 ± 4.91
Married	1969 (85.9)	54.73 ± 6.47	24.02 ± 3.22	12.52 ± 1.51	18.64 ± 2.22	64.3 ± 14.58	34.4 ± 4.7
Widowed	13 (0.6)	57.77 ± 5.8	24.31 ± 3.77	13.31 ± 0.95	19.62 ± 1.61	73.46 ± 12	37.09 ± 4.36
Divorced	99 (4.3)	55.48 ± 6.02	24.39 ± 3.45	12.73 ± 1.59	18.99 ± 2.14	66.04 ± 13.63	35.06 ± 4.6
*p*		<0.001***	0.001**	<0.001***	0.020*	0.029*	0.002**
Education							
Junior college and below	400 (17.4)	53.56 ± 7.13	23.49 ± 3.58	12.38 ± 1.67	18.16 ± 2.49	63.22 ± 15.14	33.72 ± 5.06
Undergraduates	1,191 (51.9)	54.42 ± 6.56	23.97 ± 3.24	12.51 ± 1.49	18.6 ± 2.28	64.22 ± 14.45	34.3 ± 4.72
Master/PhD	702 (30.6)	55.5 ± 5.94	24.2 ± 3.07	12.55 ± 1.51	18.94 ± 1.98	65.13 ± 14.19	34.81 ± 4.49
*p*		<0.001***	0.018*	0.485	<0.001***	0.134	0.004**
Political status							
CPC Member	1,266 (55.2)	55.29 ± 6.19	24.27 ± 3.16	12.62 ± 1.48	18.85 ± 2.11	65.51 ± 14.3	34.85 ± 4.59
Democratic	56 (2.4)	55.18 ± 5.9	23.75 ± 3	12.57 ± 1.29	18.79 ± 2.03	63.14 ± 14.75	34.27 ± 4.5
League Member	129 (5.6)	53.66 ± 6.76	23.78 ± 3.29	12.45 ± 1.55	18.4 ± 2.46	65.17 ± 13.56	34.21 ± 4.79
Masses	842 (36.7)	53.68 ± 6.85	23.52 ± 3.37	12.33 ± 1.6	18.31 ± 2.38	62.49 ± 14.76	33.64 ± 4.84
*p*		<0.001***	<0.001***	<0.001***	<0.001***	<0.001***	<0.001***
Profession							
Doctor	586 (25.6)	55.41 ± 6.04	24.13 ± 3.09	12.47 ± 1.53	18.93 ± 2.02	64.72 ± 14.26	34.69 ± 4.49
Nurse	748 (32.6)	54.37 ± 6.86	23.99 ± 3.37	12.57 ± 1.53	18.55 ± 2.39	65.6 ± 14.37	34.53 ± 4.92
Administrator	299 (13.0)	55.3 ± 6.07	24.33 ± 3.13	12.75 ± 1.33	18.9 ± 2.12	65.16 ± 14.23	34.83 ± 4.45
Multi-role	74 (3.2)	55.65 ± 5.07	24.26 ± 2.72	12.76 ± 1.4	19.19 ± 1.66	65.22 ± 13.59	34.96 ± 3.72
Other	586 (25.6)	53.61 ± 6.74	23.52 ± 3.37	12.28 ± 1.61	18.22 ± 2.32	61.75 ± 14.89	33.47 ± 4.84
*p*		<0.001***	0.003**	<0.001***	<0.001***	<0.001***	<0.001***
Service year							
Under 5 years	148 (6.5)	54.59 ± 6.21	23.72 ± 3.34	12.41 ± 1.56	18.81 ± 2.09	67.32 ± 13.06	34.85 ± 4.51
5–9 years	243 (10.6)	53.62 ± 7.18	23.58 ± 3.64	12.23 ± 1.75	18.28 ± 2.52	61.99 ± 15.26	33.52 ± 5.25
10–19 years	623 (27.2)	53.87 ± 6.82	23.65 ± 3.3	12.32 ± 1.62	18.41 ± 2.39	63.47 ± 14.47	33.9 ± 4.88
20–29 years	730 (31.8)	55.03 ± 6.12	24.1 ± 3.19	12.59 ± 1.43	18.75 ± 2.11	64.74 ± 14.41	34.59 ± 4.57
30 years or above	549 (23.9)	55.32 ± 6.3	24.35 ± 3.07	12.72 ± 1.4	18.81 ± 2.12	64.97 ± 14.53	34.78 ± 4.47
*p*		<0.001***	0.001**	<0.001***	0.015*	0.003**	0.001**
Professional title							
No title	183 (8.0)	53.66 ± 7.11	23.49 ± 3.64	12.26 ± 1.67	18.21 ± 2.52	62.49 ± 15.45	33.6 ± 5.06
The lowest title	27 (1.2)	56.26 ± 5.1	24.41 ± 3.08	12.93 ± 1.11	19.07 ± 2.29	67.37 ± 12.64	35.52 ± 3.56
Primary title	490 (21.4)	53.72 ± 6.84	23.76 ± 3.3	12.34 ± 1.63	18.3 ± 2.46	63.46 ± 14.78	33.87 ± 4.99
Mid-level title	835 (36.4)	54.23 ± 6.62	23.75 ± 3.42	12.46 ± 1.54	18.57 ± 2.22	63.66 ± 14.61	34.09 ± 4.81
Vice-senior title	423 (18.4)	55.25 ± 6.02	24.22 ± 2.9	12.55 ± 1.46	18.83 ± 2.07	65.27 ± 13.83	34.77 ± 4.32
Senior title	335 (14.6)	56.41 ± 5.64	24.67 ± 2.9	12.86 ± 1.3	19.18 ± 1.86	66.79 ± 13.96	35.51 ± 4.25
*P*		<0.001***	<0.001***	<0.001***	<0.001***	0.002**	<0.001***
Weekly working hours							
Under 40 h	1,080 (47.1)	54.56 ± 6.61	24.04 ± 3.34	12.49 ± 1.57	18.61 ± 2.28	65.43 ± 14.39	34.55 ± 4.84
41–60 h	1,124 (49.0)	54.57 ± 6.44	23.87 ± 3.21	12.49 ± 1.49	18.63 ± 2.23	63.41 ± 14.42	34.17 ± 4.6
Over 61 h	89 (3.9)	55.56 ± 6.25	24.11 ± 2.97	12.72 ± 1.49	18.82 ± 2.04	62.43 ± 16.03	34.34 ± 4.7
*P*		0.376	0.332	0.320	0.844	0.002**	0.114
Monthly income (RMB)							
Under 5,000	55 (2.4)	53.29 ± 6.97	23.04 ± 4.01	12.36 ± 1.54	17.84 ± 2.61	56.33 ± 18.41	32.28 ± 5.32
5,001–7,000	259 (11.3)	53.68 ± 7.24	23.63 ± 3.48	12.36 ± 1.65	18.19 ± 2.61	61.41 ± 15.65	33.45 ± 5.11
7,001–9,000	386 (16.8)	53.69 ± 6.8	23.53 ± 3.42	12.36 ± 1.66	18.32 ± 2.31	62.48 ± 14.31	33.65 ± 4.84
9,001–11,000	435 (19.0)	54.14 ± 6.57	23.69 ± 3.38	12.43 ± 1.57	18.51 ± 2.32	64.01 ± 14.41	34.11 ± 4.77
11,001–13,000	381 (16.6)	54.6 ± 6.33	24.06 ± 3.06	12.48 ± 1.44	18.62 ± 2.14	64.93 ± 13.57	34.47 ± 4.49
13,001–15,000	287 (12.5)	55.12 ± 5.84	24.12 ± 3.2	12.6 ± 1.44	18.74 ± 2.14	65.28 ± 14.44	34.71 ± 4.6
15,001–20,000	271 (11.8)	55.52 ± 6.03	24.28 ± 3.14	12.59 ± 1.53	19.1 ± 1.9	66.71 ± 13.7	35.15 ± 4.46
Over 20,000	219 (9.6)	56.77 ± 5.91	25.07 ± 2.43	12.88 ± 1.25	19.4 ± 1.76	68.37 ± 13.35	36 ± 3.97
*p*		<0.001***	<0.001***	0.007**	<0.001***	<0.001***	<0.001***
Turnover intention							
All the time	14 (0.6)	46.29 ± 9.6	20.21 ± 4.79	11.79 ± 1.93	16.64 ± 2.47	40.5 ± 18.93	27.03 ± 3.75
Often	61 (2.7)	48.67 ± 8.24	21.39 ± 3.5	11.46 ± 2.03	16.46 ± 2.95	44.36 ± 13.03	28.37 ± 4.37
Sometimes	498 (21.7)	51.6 ± 6.45	22.32 ± 3.35	11.91 ± 1.64	17.7 ± 2.36	54.65 ± 13.09	31.35 ± 4.27
Seldom	497 (21.7)	53.33 ± 5.96	23.37 ± 2.84	12.19 ± 1.48	18.29 ± 2.13	62.39 ± 12.38	33.48 ± 4.01
Barely	513 (22.4)	55.28 ± 5.72	24.36 ± 2.88	12.59 ± 1.41	18.82 ± 2.05	66.57 ± 12.18	35.04 ± 4.05
Not at all	710 (31.0)	57.79 ± 5.44	25.52 ± 2.81	13.16 ± 1.2	19.6 ± 1.79	73.03 ± 11.47	37.23 ± 3.84
*p*		<0.001***	<0.001***	<0.001***	<0.001***	<0.001***	<0.001***

AM, achievement motivation; SE, self-efficacy; CS, conscientiousness; GL, gratitude level; POS, perceived organizational support; ID, intrinsic driving.*p < 0.05, **p < 0.01, ***p < 0.001.

The ID and five-dimensional levels were expressed as the mean ± standard deviation. *K-W* test results showed no significant differences in ID by gender (*p* > 0.05). ID and its dimensions differed significantly among subjects of different ages (*p* < 0.05), except for POS (*p* > 0.05). There were statistically significant differences in AM and GL among subjects with varying levels of education (*p* < 0.001). In addition, ID and its five dimensions differed significantly by marital status, political status, profession, service year, professional title, monthly income, and turnover intention (*p* < 0.05). Only POS differed significantly by working hours (Table 1).

**Figure 1:**
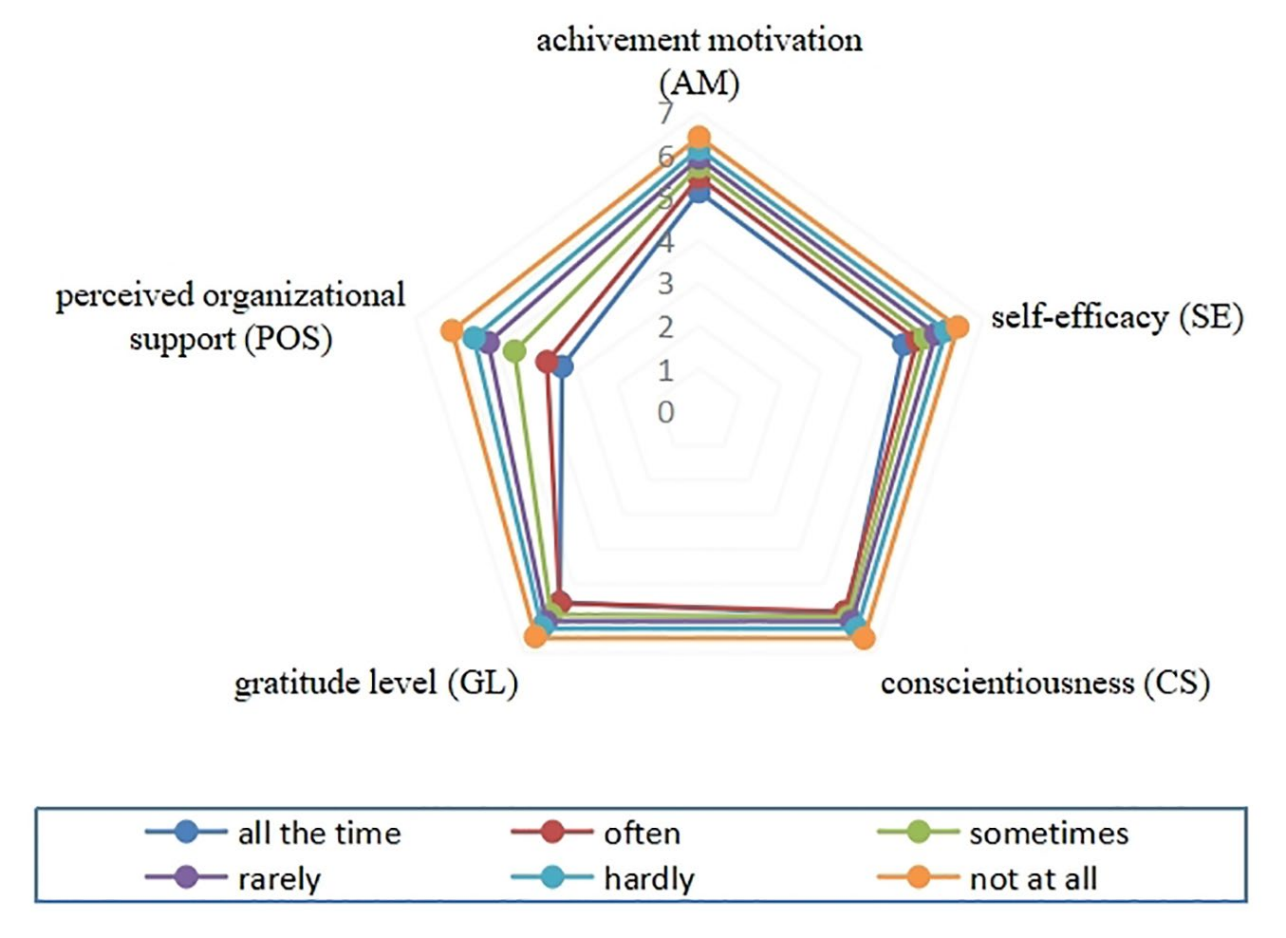
Radar chart of intrinsic drive level of people with different turnover intention.

The average of each ID dimension was calculated by dividing the total score by the number of questions in that dimension. The five dimensions differed by turnover intention with higher ID scores in the outer than inner circle (Figure 1). Individuals with a high turnover intention were mostly concentrated in the inner circle. The POS of people who wanted to leave work “all the time” was significantly lower than the POS of the group that had “no intention of leaving at all.”

### 3.2. Confirmatory factor analysis

This study selects standardized factor loadings, combined reliability and mean extracted variance for confirmatory factor analysis, and the results are shown in Supplementary Table 1. Based on the criteria proposed by Fornell and Lacker (1981), Nunnally and Bernstein (1994), and Hair et al. (1998) for convergent validity, all the standardized factor loading in this study ranged from 0.647 to 0.878, the average variance extracted is higher than 0.50, and the composite reliability is higher than 0.60 (*p* < 0.001), showing that the dimensions of the scale have good consistency.

The model fit is demonstrated by *χ*^2^, *df*, RMSEA, SRMR, GFI, NFI, TLI, CFI, and AGFI, and the result shows that the RMSEA, SRMR, GFI, NFI, TLI, CFI, and AGFI values are ideal and the *χ*^2^*/df* is acceptable (Table 2).

**Table 2:** Indicators of model fit.

Model fit	Criteria	Model fit of research model	Result
Chi-square		1391.954	
*χ*^2^/df	<5	4.63	Acceptable
RMSEA	<0.08	0.040	Ideal
SRMR	<0.08	0.0302	Ideal
GFI	>0.9	0.960	Ideal
NFI	>0.9	0.975	Ideal
TLI	>0.9	0.971	Ideal
CFI	>0.9	0.980	Ideal
AGFI	>0.9	0.938	Ideal

### 3.3. Multiple linear regression analysis

Dummy variables were created for the marital status, political status, and profession variables with the single, masses, and other professions serving as the reference, respectively (Table 3). Multiple linear regression analysis was performed for AM, and the final multiple linear model was statistically significant without severe multicollinearity (*F* = 6.078, *VIF* < 5, *p* < 0.001). The regression coefficients for marital status, political status, profession, and monthly income were 0.241–3.856 (adjusted *R*^2^ = 0.035, *p* < 0.05). The AM was stronger for married, widowed, and divorced than unmarried people. Nurses and administrators had a stronger AM than people in other professions, as did those with a higher monthly income. The regression coefficient of widowed was >3, indicating that this had the strongest influence on the AM (Table 4). The *t* value and 95% *CI* of each variable are shown in Supplementary Table 2.

**Table 3:** Variable assignments.

Variable	Assignments
*Age*	1 = under 30，2 = 31–40，3 = 41–50，4 = over 50
Marital status	
Single	0 = no，1 = yes
Married	0 = no，1 = yes
Widowed	0 = no，1 = yes
Divorced	0 = no，1 = yes
*Education*	1 = junior college and below，2 = undergraduates, 3 = Master/PhD
Political status	
CPC Members	0 = no，1 = yes
Democratic	0 = no，1 = yes
League Member	0 = no，1 = yes
Masses	0 = no，1 = yes
Profession	
Doctor	0 = no，1 = yes
Nurse	0 = no，1 = yes
Administrator	0 = no，1 = yes
Multi-role	0 = no，1 = yes
Other	0 = no，1 = yes
*Service year*	1 = under 5 years，2 = 5–9 years，3 = 10–19 years，4 = 20–29 years， 5 = 30 years and above
*Professional title*	1 = no title，2 = the lowest title，3 = primary title，4 = mid-level title，5 = vice-senior title，6 = senior title
*Weekly working hours*	1 = under 40 h，2 = 41–60 h，3 = over 61 h
*Monthly income (RMB)*	1 = under 5,000，2 = 5,001–7,000，3 = 7,001–9,000, 4 = 9,001–11,000， 5 = 11,001–13,000，6 = 13,001–15,000，7 = 15,001–20,000，8 = over 20,000

**Table 4:** Regression coefficient of intrinsic drive and its five dimensions.

	AM	SE	CS	GL	POS	ID
*Constant*	50.334***	21.944***	11.541***	17.657***	61.83***	32.166***
*Age*	0.216	0.097	−0.006	0.09	0.838	0.227
Marital status						
Married	1.396**	0.674*	0.379**	0.187	0.874	0.58
Widowed	3.856*	0.635	0.912*	0.856	7.32	2.611
Divorced	2.061*	1.111**	0.592**	0.634*	2.681	1.316*
Single	–	–	–	–	–	–
*Education*	0.264	0.175	0.027	0.102	−0.462	0.064
Political status						
CPC Members	1.072***	0.526**	0.145*	0.285**	2.47***	0.844***
Democratic	1.024	−0.212	0.06	0.132	−0.418	0.054
League Member	1.275*	0.818*	0.365*	0.21	1.331	0.794
Masses	–	–	–	–	–	–
Profession						
Doctor	0.867	0.187	−0.02	0.306*	0.982	0.418
Nurse	0.785*	0.406*	0.221**	0.38**	3.951***	1.05***
Administrator	1.018*	0.268	0.278**	0.416**	1.236	0.55
Multi-role	1.059	0.31	0.249	0.573*	1.805	0.872
Other	–	–	–	–	–	–
*Service year*	−0.076	0.043	0.039	−0.095	−1.638**	−0.292
*Professional title*	0.036	−0.066	0.069	−0.033	−0.035	−0.018
*Weekly working hours*	−0.218	−0.235	−0.02	−0.094	−2.272***	−0.562**
*Monthly income (RMB)*	0.241**	0.152**	0.015	0.134***	1.457***	0.384***

AM, achievement motivation; SE, self-efficacy; CS, conscientiousness; GL, gratitude-level; POS, perceived organizational support; ID, intrinsic driving.

*p < 0.05, **p < 0.01, ***p < 0.001.

Multiple linear regression analysis was performed for SE, and the final multiple linear model was statistically significant without severe multicollinearity (*F* = 4.846, *VIF* < 5, *p* < 0.001). The regression coefficients for marital status, political status, profession, and monthly income were 0.152–1.111 (adjusted *R*^2^ = 0.026, *p* < 0.05). The level of self-efficacy was higher for married and divorced people than single people, higher for CPC and Communist Youth League members than the masses, higher for nurses than people in other professions, and higher for those with a higher monthly income (Table 4). The *t* value and 95% *CI* of each variable are shown in Supplementary Table 3.

The multilinear model constructed by regression analysis of CS was statistically significant, without severe multicollinearity (*F* = 5.228, *VIF* < 5, *p* < 0.001). Similar to SE, CS was higher for married and divorced people than single people, CPC and Communist Youth League members than the masses, and nurses and administrators than people in other professions (adjusted *R^2^* = 0.029, *p* < 0.05). However, CS was not impacted by monthly income (Table 4). The *t* value and 95% CI of each variable are shown in Supplementary Table 4.

The regression analysis of GL was statistically significant without multicollinearity (*F* = 6.068, *VIF* < 5, *p* < 0.001). The influencing factors included marital status, political status, profession, and monthly income, all of which had positive effects (Table 4) (adjusted *R^2^* = 0.035, *p* < 0.05). The *t* value and 95% *CI* of each variable are shown in Supplementary Table 5.

The multilinear model of POS was statistically significant and lacked severe multicollinearity (*F* = 8.411, *VIF* < 5, *p* < 0.001). Political status, profession, service year, weekly working hours, and monthly income were the influencing factors with regression coefficients ranging from −2.272 to 3.951 (adjusted *R*^2^ = 0.049, *p* < 0.05). Respondent POS was higher for CPC members, nurses, and those with a higher monthly income, and lower for individuals with higher service years and more working hours per week. The *t* value and 95% *CI* of each variable are shown in Supplementary Table 6. The regression coefficients of these factors were all >1, with the coefficients for nurses near 4 and the coefficients for CPC members and weekly working hours all >2, indicating that these values had a large impact on POS (Table 4).

Multiple regression analysis results suggested that positive influencing factors of ID included divorce, CPC membership, being a nurse, working a lower number of hours per week, and having a higher monthly income (*F* = 7.248, *p* < 0.001, adjusted *R^2^* = 0.042) (Table 4). The *t* value and 95% *CI* of each variable are shown in Supplementary Table 7.

### 3.4. Correlation analysis

**Figure 2:**
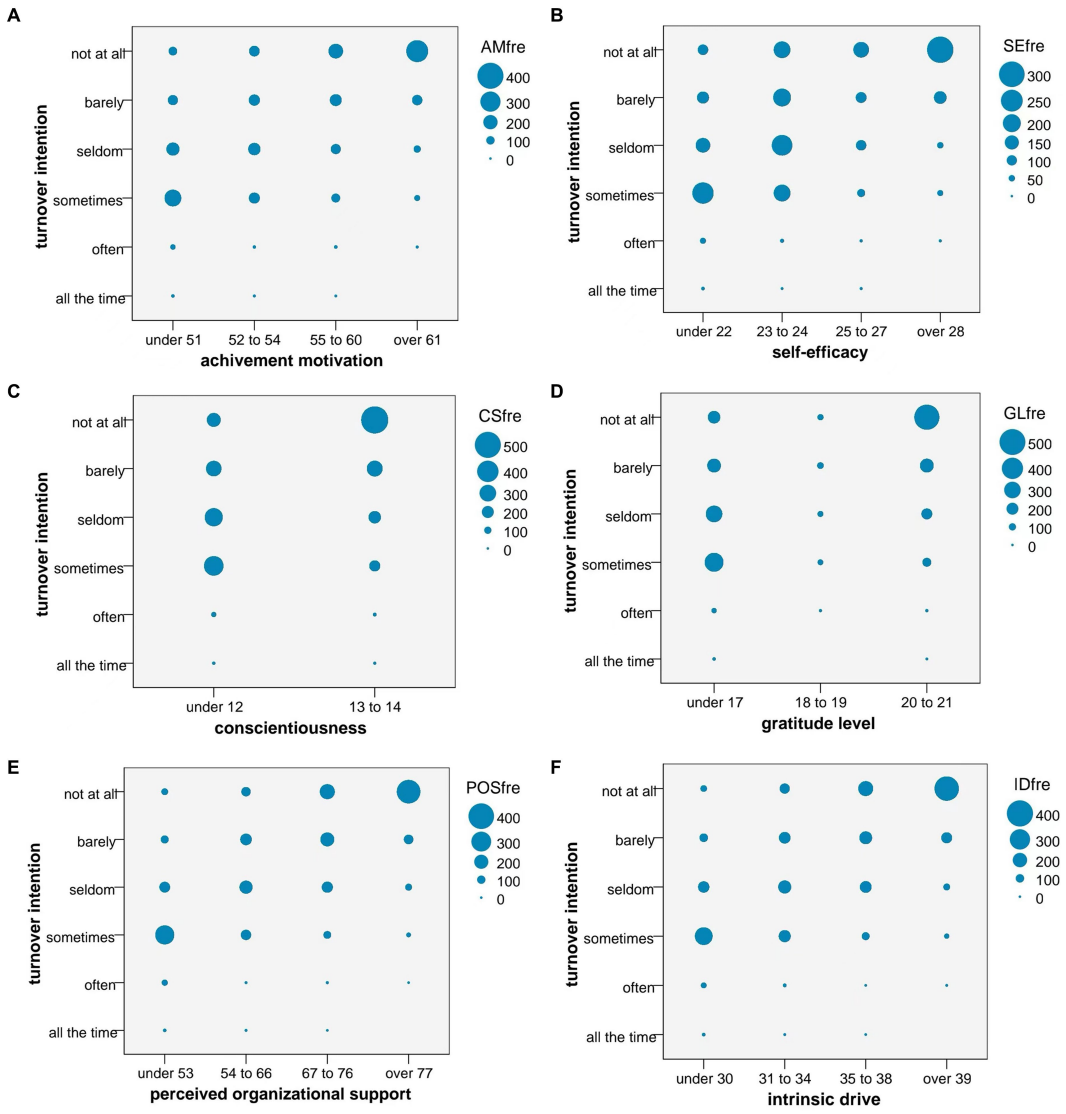
**(A)** The number of people with varying levels of turnover intention in achievement motivation. AMfre = Frequency of different turnover intentions at different achievement motivation levels. **(B)** The number of people with varying levels of turnover intention in self-efficacy. SEfre = Frequency of different turnover intentions at different self-efficacy levels. **(C)** The number of people with varying levels of turnover intention in conscientiousness. CSfre = Frequency of different turnover intentions at different conscientiousness levels. Because the scores for conscientiousness differed little, they were divided into two groups. **(D)** The number of people with varying levels of turnover intention in gratitude level. GLfre = Frequency of different turnover intentions at different gratitude level levels. Because the scores for gratitude level differed little, they were divided into three groups. **(E)** The number of people with varying levels of turnover intention in perceived organizational support. POSfre = Frequency of different turnover intentions at different perceived organizational support levels. **(F)** The number of people with varying levels of turnover intention in intrinsic drive. IDfre = Frequency of different turnover intentions at different intrinsic drive levels.

Intrinsic drive and its five dimensions were grouped by quartile to form ordered categorical variables. Kendall’s tau-b rank correlation coefficient was used to evaluate whether there was a correlation between ID and the turnover intention of medical staff. The original motivation data were also used to determine if there was a correlation between ID and turnover intention using Spearman rank correlation analysis. Both methods showed that AM, SE, CS, GL, POS, and ID correlated positively with turnover intention (*p* < 0.001, Table 5). The number of people with varying levels of turnover intention in each dimension of ID is shown in Figure 2. The dot size represents the frequency with which turnover intention was chosen. When drive and its dimensions scores were low, people tended to choose “sometimes want to turnover” and as drive increased, more people chose “never want to turnover.”

**Table 5:** Correlation analysis of intrinsic drive and turnover tendency.

Variables	Kendall’s tau-b	Spearman’s rho
AM	0.324***	0.406***
SE	0.338***	0.410***
CS	0.265***	0.348***
GL	0.296***	0.365***
POS	0.432***	0.522***
ID	0.414***	0.514***

AM, achievement motivation; SE, self-efficacy; CS, conscientiousness; GL, gratitude level; POS, perceived organizational support; ID, intrinsic driving.

***p < 0.001.

## 4. Discussion

### 4.1. Influence of sociodemographic factors on intrinsic drive

This study fills a knowledge gap by gathering data on the intrinsic motivation of medical personnel. The findings offer a foundation to inspire medical staff from a sociodemographic perspective and boost their work motivation. The use of self-designed questionnaires made the measurement instrument more relevant, and quality control ensured the validity and authenticity of the data. The study identified differences in the level ID by marital status, political status, profession, service year, number of working hours per week, and monthly income. There was also a correlation between ID and turnover intention. Individuals with a stronger drive had a weaker turnover intention.

Given the low ratio of male to female health workers in China (1:2.6) and the universality of the population distribution, it is critical to assess ID by gender. The findings showed no difference in the intrinsic drive of males and females. While male and female medical staff are physically distinct, they appeared to have the same love for the industry and sense of responsibility for their jobs. The study findings indicated that marital status and political status differed at each ID dimension. Married, divorced, and widowed people performed better in all dimensions than single people, particularly in AM. A 2019 survey of 144 Chinese hospitals found that married and widowed medical staff had lower self-rated health scores than unmarried people (Wu, 2020). The former had experienced hard times emotionally and their depression may have contributed to a decline in health. At the same time, these individuals tended to put more energy into their work. It is possible, however, that the results are biased due to an uneven distribution of the population. While it is important to pay close attention to the health status of married and widowed staff, it is also critical to focus on the needs and appeals of unmarried people. If necessary, hospitals should provide appropriate ways for single employees to make friends and improve their living environment, stimulating their ID.

There is a noteworthy difference between the masses and CPC members. The *β* of CPC members was 1.072 (*p* < 0.001) for the AM dimension, with the masses serving as the reference, suggesting that achievement motivation was significantly higher for CPC members than for the masses. Similar findings were found in ID and the other four dimensions, especially in POS (*β* = 2.47, *p* < 0.001). This may be related to how the CPC fosters its members, suggesting that it may be worth using a similar approach to increase the ID of medical staff and motivate them to offer great medical treatment. This could be accomplished by promoting the noble sentiments of medical staff to save lives or using the power of good role models to encourage medical workers to be optimistic and execute their jobs well. Learning and comprehending a hospital’s development process and culture may improve its emotional integration and cohesiveness. Employees can be effectively cared for by paying close attention to their needs and changing perspectives (Yang and Maresova, 2021).

### 4.2. Influence of work-related factors on intrinsic drive

This study found differences in the ID of individuals from various professions. Employees in “other” professions, such as pharmacists and technicians, had lower ID than doctors, nurses, administrators, and multi-role staff. Doctors and nurses, as front-line caregivers, often receive more support and attention from both institutions and patients while people in “other” professions may feel underappreciated and undervalued (Lv, 2020). The absence of ID or an active job may make it a challenge for personnel in “other” positions to be subjective and provide strong support and medical services. Medical institutions should be aware that each job has value and pay attention to people in various roles, providing affirmation and support, and fully exploring the work potential of every staff person. Knowledge training provided during epidemic prevention can allow nurses to practice and acquire skills that contribute to a sense of self-efficacy (Fan et al., 2020; Santos, 2020). Policymakers and administrators should actively guide and motivate medical staff to gain competence and progress professionally, which may in turn improve their ID.

This study identified significant differences in ID and its five dimensions by the number of service years. Except for those who had worked for ≤5 years, more service years correlated with a higher ID (Table 1). Nonetheless, regression analysis of the POS dimension had a negative coefficient (−1.638), implying that POS decreases as the number of service years increases. This could be related to the unique characteristics of people who have worked for ≤5 years. As new employees, these individuals often receive more attention and support from the hospital and have greater resources and platforms than long-term employees, resulting in higher POS. A cut-off point occurs at 5 years, after which the level of POS declines and then gradually increases with the accumulation of work experience and service years. This matches the findings of a 2016 survey. The level of organizational support for medical staff with 1–5, 6–15, and > 16 years of experience were 72.0 (57.0, 82.0), 71.0 (56.0, 81.0) and 74.0 (63.0, 85.8), respectively [*M*(P_25_, P_75_), *p* < 0.01] (Wu et al., 2019). Employees who have worked for ≤5 years may receive more guidance and help while those who have worked for 15–20 years have often made particular achievements and gained status, both of which are a focus of the hospital’s attention that is not provided to other workers. Thus, hospitals should consider whether they are paying enough attention to employees with 6–19 service years, including whether these individuals are being given appropriate development tools and opportunities, and whether their ideas and suggestions are valued. If not, then necessary changes should be made in those areas.

With the exception of staff with the lowest title, those with a higher title had a higher intrinsic drive (Table 1). Individuals with a higher title have more responsibility and influence, which can motivate them to be more active in their workplace. The enthusiasm for work promotes greater results and increases the opportunity to receive a higher professional title. More work hours per week had a significantly negative impact on POS. Doctors in China tend to have a heavy workload, especially in grade-A tertiary hospitals where doctors often need to work overtime to complete various non-medical tasks. This is clearly detrimental to the drive of medical staff. Administrators should consider lightening the load of medical staff, potentially by removing some of the clutter of clerical work (Shanafelt et al., 2016). In addition, the association between monthly income and ID suggests that the salary of medical staff should be increased, along with greater affirmation of their work achievements.

### 4.3. Correlation between intrinsic drive and turnover intention

A 2018 survey of Chinese medical staff found that more than half had a high or medium turnover intention. Turnover intention is shown to be a strong predictor of final turnover behavior (Hamidi et al., 2018). For example, a domestic survey found that of medical staff who were willing to leave, 43.7% wanted to leave the health industry (Liu et al., 2017). Turnover intention not only affects economic benefits but also influences patient satisfaction, organizational effectiveness, and public relations (Wang, 2019). One study found that turnover and temporary replacement costs in Australia, the US, Canada, and New Zealand were $46,790, $20,561, $26,652, and $23,711 per caregiver, respectively (Duffield et al., 2014). Resignation not only reduces talent but also causes financial losses and can even damage patient health. The correlation analysis conducted in the current study showed that ID and its five dimensions were positively correlated with turnover intention, of which the correlation between POS and turnover intention was the most strong. Turnover intention was assigned values ranging from 1 to 6, from “want to leave at all times” to “do not want to leave at all.” Higher ID correlated with lower turnover intention, highlighting the importance of stimulating the ID of medical staff.

### 4.4. Potential strategies for stimulating intrinsic drive

The book “Contradication” stated that “external causes work through internal causes.” The main strategy to modify external performance was to resolve the intrinsic difficulties of medical professionals. Incentives to stimulate medical staff intrinsic drive and foster a good practice environment from the outside can be an effective way to both relieve psychological pressure and improve the practice status of medical staff. Common intervention methods are recommended that combine psychological expertise and the characteristics of ID. Policymakers should establish a favorable environment to improve doctor-patient interactions and develop multi-dimensional social support to positively impact medical staff motivation (Li et al., 2022). Administrators may help lead cognitive assessments by conducting group counseling and psychology lectures (Bao et al., 2020; Wang et al., 2021; Wan et al., 2022), implementing cognitive behavioral and organizational behavioral treatments focused on mindfulness (Hammer et al., 2015; David et al., 2018; Harolds, 2020a; Naser et al., 2021), and assisting employees to keep a healthy work-life balance to improve their work concentration. Psychological therapy and mental health classes can also help medical students to develop decompression skills, self-management tactics, and the capacity to deal with obstacles (Duprez et al., 2017; Harolds, 2020b). Psychological intervention trials may be used to investigate the benefits of techniques and strategies aimed at promoting the ID of medical staff.

## 5. Limitations

This study has some limitations. First, the survey respondents came from 22 Beijing-affiliated hospitals, all of which were tertiary hospitals or above. The specificity of these hospital administrations and the particular geographical features of Beijing may have led to a biased selection of respondents that is not reflective of all medical staff. While prior studies of the same 22 hospitals have shown that this selection is feasible and scientific (Chen et al., 2022) and the large sample size of >2,000 respondents helped to increase the representativeness of the current study, potential bias cannot be ruled out. Subsequent studies will need to include primary care institutions, other hospital types, and additional geographic areas, to gain a more comprehensive understanding of the ID of medical staff. Second, studies found that organizational support influences employees’ work attitudes and behaviors by acting on different aspects of their motivation (Ling et al., 2006). The sense of organizational support is similar to the need for belonging in self-determination theory, and the satisfaction of the need for belonging can significantly enhance the intrinsic motivation of individuals (Deci and Ryan, 2000; Fang et al., 2018). While POS is a strong predictor of ID, there is still controversy about whether to include POS in ID models. More research is required to explore and determine whether it needs to be replaced with other factors. Third, this study did not propose the interventions aimed to improve the ID. More professional techniques and means are needed to develop realistic methods for improving the ID of medical professionals. Since research on the ID of medical staff is still in its infancy, this study primarily focused on assessing the influence of sociodemographic characteristics. The role of individual personality traits, social support, and receipt of mental health services should also be investigated. Longitudinal experimental studies are needed to investigate in-depth effective psychological interventions and establish incentive mechanisms for medical staff.

## 6. Conclusion

Anxiety, depression, turnover intention, and burnout are increasing among medical staff. Appropriate interventions are needed to stimulate employee intrinsic drive, combine with motivation from the outside can help to provide a good practice environment and atmosphere. These may offer an efficient way to relieve psychological pressure and optimize the practice status of medical staff. This study found that marital status, political status, profession, service year, weekly working hours, and monthly income can all influence the ID of medical staff. This study identified a correlation between ID and turnover intention, indicating that stimulating ID may help to increase job retention. Future studies are needed to develop specific interventions aimed at increasing ID and providing options for medical institutions to support their staff.

## Data availability statement

The raw data supporting the conclusions of this article will be made available by the authors, without undue reservation.

## Ethics statement

The studies involving human participants were reviewed and approved by Medical Ethics Committee of Capital Medical University. Written informed consent for participation was not required for this study in accordance with the national legislation and the institutional requirements.

## Author contributions

YZ and JL: concept and design. YZ, ZY, CW, and JL: data collection. YZ and ZY: analysis and interpretation of data. YZ and TC: draft manuscript. YZ, TC, CW, and JL: critical revision of the manuscript for important intellectual content. CW and JL: acquired funding. All the authors critically revised the manuscript drafts and approved the submission.

## Funding

This study was funded by grant from Beijing Municipal Social Science Foundation (21JCC116) and grant from Capital Research Base of Health Management and Policy (2021JD04).

## Conflict of interest

The authors declare that the research was conducted in the absence of any commercial or financial relationships that could be construed as a potential conflict of interest.

## Publisher’s note

All claims expressed in this article are solely those of the authors and do not necessarily represent those of their affiliated organizations, or those of the publisher, the editors and the reviewers. Any product that may be evaluated in this article, or claim that may be made by its manufacturer, is not guaranteed or endorsed by the publisher.
