# Supplementary material for: Intrinsic drive of medical staff: a survey of employee representatives from 22 hospitals in China
Source: Front Psychol. 2023 Apr 27;14:1157823. doi: 10.3389/fpsyg.2023.1157823 (PMC10172483; doi:10.3389/fpsyg.2023.1157823)
Supplement: Supplementary file 1 [file Data_Sheet_1.docx]

Supplementary Material

Intrinsic drive of medical staff: a survey of employee representatives from 22 hospitals in China

Yuqing Zhang, Zheng Yuan, Taozhu Cheng, Cunliang Wang, Jun Li^*^

*** Correspondence:** Jun Li: richardl@ccmu.edu.cn

# The names of the 22 investigated hospitals

1. Beijing Friendship Hospital, Capital Medical University
2. Beijing Tongren Hospital, Capital Medical University
3. Beijing Chao-yang Hospital, Capital Medical University
4. Beijing Jishuitan Hospital
5. Beijing Tiantan Hospital, Capital Medical University
6. Beijing Anzhen Hospital, Capital Medical University
7. Beijing Shijitan Hospital, Capital Medical University
8. Xuanwu Hospital Capital Medical University
9. Beijing Hospital of Traditional Chinese Medicine, Capital Medical University
10. Beijing Cancer Hospital
11. Beijing Children’s Hospital, Capital Medical University
12. Children’s Hospital, Capital Institute of Pediatrics
13. Beijing Obstetrics and Gynecology Hospital, Capital Medical University
14. Beijing Stomatological Hospital, Capital Medical University
15. Beijing Chest Hospital, Capital Medical University
16. Beijing Youan Hospital, Capital Medical University
17. Beijing Ditan Hospital, Capital Medical University
18. Beijing Anding Hospital, Capital Medical University
19. Beijing Huilongguan Hospital
20. Beijing Xiaotangshan Hospital
21. Beijing Geriatric Hospital
22. Beijing Tsinghua Changgung Hospital

# The flowchart for building the scale


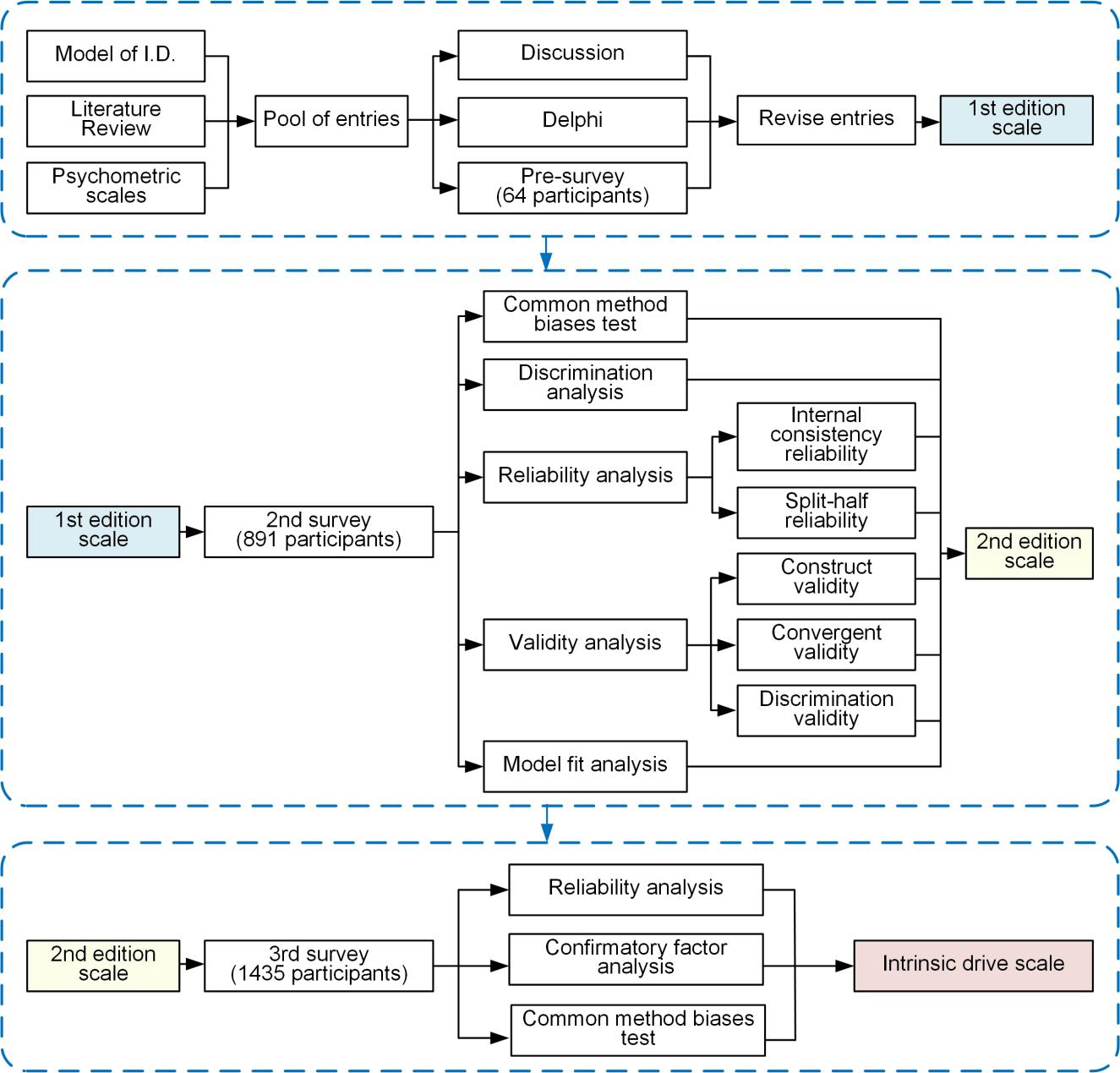


ID, intrinsic drive.

# Supplementary Tables-Detailed statistical analysis results

|  | **standardized factor loading** | **average variance extracted** | **composite reliability** |
| --- | --- | --- | --- |
| AM1<---AM | 0.684 | 0.505 | 0.901 |
| AM2<---AM | 0.779 |  |  |
| AM3<---AM | 0.753 |  |  |
| AM4<---AM | 0.744 |  |  |
| AM5<---AM | 0.676 |  |  |
| AM6<---AM | 0.733 |  |  |
| AM7<---AM | 0.647 |  |  |
| AM8<---AM | 0.688 |  |  |
| AM9<---AM | 0.679 |  |  |
| SE1<---SE | 0.765 | 0.619 | 0.867 |
| SE2<---SE | 0.796 |  |  |
| SE3<---SE | 0.815 |  |  |
| SE4<---SE | 0.771 |  |  |
| CS1<---CS | 0.865 | 0.734 | 0.846 |
| CS2<---CS | 0.848 |  |  |
| GL1<---GL | 0.847 | 0.553 | 0.786 |
| GL2<---GL | 0.693 |  |  |
| GL3<---GL | 0.679 |  |  |
| POS1<---POS | 0.795 | 0.675 | 0.961 |
| POS5<---POS | 0.854 |  |  |
| POS6<---POS | 0.828 |  |  |
| POS7<---POS | 0.778 |  |  |
| POS8<---POS | 0.856 |  |  |
| POS9<---POS | 0.878 |  |  |
| POS10<---POS | 0.697 |  |  |
| POS11<---POS | 0.863 |  |  |
| POS12<---POS | 0.876 |  |  |
| POS2<---POS | 0.838 |  |  |
| POS3<---POS | 0.798 |  |  |
| POS4<---POS | 0.779 |  |  |

AM,achievement motivation; SE,self-efficacy; CS,conscientiousness; GL,gratitude-level; POS,perceived organizational support.

**TABLE 2 Analysis of Influencing Factors of Achievement Motivation**

|  | ***B*** | ***t*** | **95% *CI*** | ***P*** |
| --- | --- | --- | --- | --- |
| Constant | 50.334 | 58.066 | （48.634，52.034） | <0.001*** |
| Age | 0.216 | 0.734 | （-0.36，0.792） | 0.463 |
| Marital status (reference variable is "Single") | | | | |
| Married | 1.396 | 2.694 | （0.38，2.411） | 0.007** |
| Widowed | 3.856 | 2.162 | （0.359，7.352） | 0.031* |
| Divorced | 2.061 | 2.57 | （0.488，3.633） | 0.010* |
| Education | 0.264 | 0.933 | （-0.291，0.818） | 0.351 |
| Political status (reference variable is "Masses") | | | | |
| CPC Member | 1.072 | 3.553 | （0.48，1.664） | <0.001*** |
| Democratic | 1.024 | 1.15 | -0.722，2.771） | 0.250 |
| League Member | 1.275 | 1.881 | （-0.054，2.604） | 0.060 |
| Profession (reference variable is "Other") | | | | |
| Doctor | 0.867 | 1.941 | （-0.009，1.743） | 0.052 |
| Nurse | 0.785 | 2.229 | （0.094，1.475） | 0.026* |
| Administrator | 1.018 | 2.192 | （0.107，1.929） | 0.028* |
| Multiple roles | 1.059 | 1.365 | （-0.463，2.581） | 0.172 |
| Service year | -0.076 | -0.314 | （-0.552，0.399） | 0.754 |
| Professional title | 0.036 | 0.224 | （-0.28，0.352） | 0.823 |
| Weekly working hours | -0.218 | -0.921 | （-0.683，0.246） | 0.357 |
| Monthly income (RMB) | 0.241 | 2.647 | （0.063，0.42） | 0.008** |

**P*<0.05, ***P*<0.01, ****P*<0.001.

**TABLE 3 Analysis of Influencing Factors of Self-efficacy**

|  | ***B*** | ***t*** | **95% *CI*** | ***P*** |
| --- | --- | --- | --- | --- |
| Constant | 21.944 | 49.569 | （21.076，22.812） | <0.001*** |
| Age | 0.097 | 0.646 | （-0.198，0.393） | 0.518 |
| Marital status (reference variable is "Single") | | | | |
| Married | 0.674 | 2.542 | （0.154，1.194） | 0.011* |
| Widowed | 0.635 | 0.693 | （-1.16，2.43） | 0.488 |
| Divorced | 1.111 | 2.696 | （0.303，1.92） | 0.007** |
| Education | 0.175 | 1.213 | （-0.108，0.458） | 0.225 |
| Political status (reference variable is "Masses") | | | | |
| CPC Member | 0.526 | 3.399 | （0.222，0.829） | 0.001** |
| Democratic | -0.212 | -0.47 | （-1.094，0.671） | 0.638 |
| League Member | 0.818 | 2.364 | （0.139，1.496） | 0.018* |
| Profession (reference variable is "Other") | | | | |
| Doctor | 0.187 | 0.816 | （-0.262，0.636） | 0.415 |
| Nurse | 0.406 | 2.246 | （0.051，0.76） | 0.025* |
| Administrator | 0.268 | 1.129 | （-0.198，0.734） | 0.259 |
| Multiple roles | 0.31 | 0.777 | （-0.472，1.091） | 0.437 |
| Service year | 0.043 | 0.342 | （-0.202，0.287） | 0.732 |
| Professional title | -0.066 | -0.802 | （-0.228，0.096） | 0.423 |
| Weekly working hours | -0.235 | -1.937 | （-0.473，0.003） | 0.053 |
| Monthly income (RMB) | 0.152 | 3.263 | （0.061，0.244） | 0.001** |

**P*<0.05, ***P*<0.01, ****P*<0.001.

**TABLE 4 Analysis of Influencing Factors of Conscientiousness**

|  | ***B*** | ***t*** | **95% *CI*** | ***P*** |
| --- | --- | --- | --- | --- |
| Constant | 11.541 | 60.362 | （11.166，11.916） | <0.001*** |
| Age | -0.006 | -0.089 | （-0.133，0.121） | 0.929 |
| Marital status (reference variable is "Single") | | | | |
| Married | 0.379 | 3.32 | （0.155，0.603） | 0.001** |
| Widowed | 0.912 | 2.329 | （0.144，1.681） | 0.02* |
| Divorced | 0.592 | 3.34 | （0.245，0.94） | 0.001** |
| Education | 0.027 | 0.434 | （-0.095，0.15） | 0.664 |
| Political status (reference variable is "Masses") | | | | |
| CPC Member | 0.145 | 2.169 | （0.014，0.276） | 0.030* |
| Democratic | 0.06 | 0.309 | （-0.321，0.441） | 0.757 |
| League Member | 0.365 | 2.449 | （0.073，0.658） | 0.014* |
| Profession (reference variable is "Other") | | | | |
| Doctor | -0.02 | -0.199 | （-0.213，0.174） | 0.842 |
| Nurse | 0.221 | 2.837 | （0.068，0.374） | 0.005** |
| Administrator | 0.278 | 2.712 | （0.077，0.479） | 0.007** |
| Multiple roles | 0.249 | 1.454 | （-0.087，0.586） | 0.146 |
| Service year | 0.039 | 0.722 | （-0.066，0.144） | 0.470 |
| Professional title | 0.069 | 1.954 | （0，0.139） | 0.051 |
| Weekly working hours | -0.02 | -0.385 | （-0.123，0.082） | 0.700 |
| Monthly income (RMB) | 0.015 | 0.753 | （-0.024，0.055） | 0.452 |

**P*<0.05, ***P*<0.01, ****P*<0.001.

**TABLE 5 Analysis of Influencing Factors of Gratitude-Level**

|  | ***B*** | ***t*** | **95% *CI*** | ***P*** |
| --- | --- | --- | --- | --- |
| Constant | 17.657 | 62.032 | （17.099，18.215） | <0.001*** |
| Age | 0.09 | 0.926 | （-0.1，0.279） | 0.355 |
| Marital status (reference variable is "Single") | | | | |
| Married | 0.187 | 1.095 | （-0.148，0.522） | 0.274 |
| Widowed | 0.856 | 1.47 | （-0.286，1.999） | 0.142 |
| Divorced | 0.634 | 2.39 | （0.114，1.154） | 0.017* |
| Education | 0.102 | 1.102 | （-0.08，0.284） | 0.271 |
| Political status (reference variable is "Masses") | | | | |
| CPC Member | 0.285 | 2.873 | （0.09，0.479） | 0.004** |
| Democratic | 0.132 | 0.457 | （-0.434，0.698） | 0.647 |
| League Member | 0.21 | 0.941 | （-0.228，0.648） | 0.347 |
| Profession (reference variable is "Other") | | | | |
| Doctor | 0.306 | 2.092 | （0.019，0.593） | 0.037* |
| Nurse | 0.38 | 3.274 | （0.152，0.607） | 0.001** |
| Administrator | 0.416 | 2.732 | （0.117，0.715） | 0.006** |
| Multiple roles | 0.573 | 2.259 | （0.076，1.07） | 0.024* |
| Service year | -0.095 | -1.187 | （-0.251，0.062） | 0.235 |
| Professional title | -0.033 | -0.615 | （-0.137，0.071） | 0.539 |
| Weekly working hours | -0.094 | -1.214 | （-0.247，0.058） | 0.225 |
| Monthly income (RMB) | 0.134 | 4.464 | （0.075，0.192） | <0.001*** |

**P*<0.05, ***P*<0.01, ****P*<0.001.

**TABLE 6 Analysis of Influencing Factors of Perceived Organizational Support**

|  | ***B*** | ***t*** | **95% *CI*** | ***P*** |
| --- | --- | --- | --- | --- |
| Constant | 61.83 | 31.663 | （58.001，65.659） | <0.001*** |
| Age | 0.838 | 1.263 | （-0.463，2.139） | 0.207 |
| Marital status (reference variable is "Single") | | | | |
| Married | 0.874 | 0.747 | （-1.419，3.167） | 0.455 |
| Widowed | 7.32 | 1.817 | （-0.581，15.221） | 0.069 |
| Divorced | 2.681 | 1.48 | （-0.87，6.233） | 0.139 |
| Education | -0.462 | -0.727 | （-1.71，0.785） | 0.467 |
| Political status (reference variable is "Masses") | | | | |
| CPC Member | 2.47 | 3.626 | （1.134，3.807） | <0.001*** |
| Democratic | -0.418 | -0.211 | （-4.303，3.466） | 0.833 |
| League Member | 1.331 | 0.873 | （-1.659，4.32） | 0.383 |
| Profession (reference variable is "Other") | | | | |
| Doctor | 0.982 | 0.974 | （-0.996，2.96） | 0.330 |
| Nurse | 3.951 | 4.968 | （2.391，5.51） | <0.001*** |
| Administrator | 1.236 | 1.183 | （-0.814，3.287） | 0.237 |
| Multiple roles | 1.805 | 1.029 | （-1.634，5.244） | 0.304 |
| Service year | -1.638 | -2.992 | （-2.712，-0.565） | 0.003** |
| Professional title | -0.035 | -0.097 | （-0.747，0.677） | 0.923 |
| Weekly working hours | -2.272 | -4.254 | （-3.32，-1.225） | <0.001*** |
| Monthly income (RMB) | 1.457 | 7.078 | （1.054，1.861） | <0.001*** |

**P*<0.05, ***P*<0.01, ****P*<0.001.

**TABLE 7 Analysis of Influencing Factors of Intrinsic Drive**

|  | ***B*** | ***t*** | **95% *CI*** | ***P*** |
| --- | --- | --- | --- | --- |
| Constant | 32.166 | 49.564 | （30.893，33.439） | <0.001*** |
| Age | 0.227 | 1.029 | （-0.205，0.658） | 0.304 |
| Marital status (reference variable is "Single") | | | | |
| Married | 0.58 | 1.492 | （-0.182，1.343） | 0.136 |
| Widowed | 2.611 | 1.948 | （-0.018，5.24） | 0.052 |
| Divorced | 1.316 | 2.183 | （0.134，2.497） | 0.029* |
| Education | 0.064 | 0.303 | （-0.351，0.479） | 0.762 |
| Political status (reference variable is "Masses") | | | | |
| CPC Member | 0.844 | 3.732 | （0.4，1.287） | <0.001*** |
| Democratic | 0.054 | 0.083 | （-1.238，1.347） | 0.934 |
| League Member | 0.794 | 1.566 | （-0.2，1.789） | 0.117 |
| Profession (reference variable is "Other") | | | | |
| Doctor | 0.418 | 1.248 | （-0.239，1.074） | 0.212 |
| Nurse | 1.05 | 3.975 | （0.532，1.567） | <0.001*** |
| Administrator | 0.55 | 1.585 | （-0.131，1.231） | 0.113 |
| Multiple roles | 0.872 | 1.494 | （-0.272，2.016） | 0.135 |
| Service year | -0.292 | -1.606 | （-0.649，0.065） | 0.108 |
| Professional title | -0.018 | -0.146 | （-0.254，0.219） | 0.884 |
| Weekly working hours | -0.562 | -3.17 | （-0.91，-0.214） | 0.002** |
| Monthly income (RMB) | 0.384 | 5.623 | （0.25，0.518） | <0.001*** |

**P*<0.05, ***P*<0.01, ****P*<0.001.

# **Intrinsic drive scale of medical staff**

Please read the following questions carefully and tick √ under the corresponding number according to the degree of agreement.

1=absolutely disagree 2=disagree 3=somewhat disagree 4=neither agree nor disagree

5=somewhat agree 6=agree 7=absolutely agree

| **Questions** | **1** | **2** | **3** | **4** | **5** | **6** | **7** |
| --- | --- | --- | --- | --- | --- | --- | --- |
| **Achivement motivation** |  |  |  |  |  |  |  |
| 1. I am willing to concentrate on my work and make unremitting efforts in order to succeed |  |  |  |  |  |  |  |
| 1. I usually make a serious review to improve my working methods when my performance is not satisfactory |  |  |  |  |  |  |  |
| 1. I feel a sense of accomplishment when I finish a job successfully, even if no one knows it |  |  |  |  |  |  |  |
| 1. I will try my best to do whatever I think is valuable, no matter what others think |  |  |  |  |  |  |  |
| 1. I pursue a good job not for the fame, but because I am interested in the work |  |  |  |  |  |  |  |
| 1. I often hold myself to a higher standard at work than others hold me to |  |  |  |  |  |  |  |
| 1. Work has its own pleasure and I am willing to do any interesting work |  |  |  |  |  |  |  |
| 1. I often forget to sleep or eat in order to finish a job I love |  |  |  |  |  |  |  |
| 1. I know exactly how hard I have to work to get where I want to be |  |  |  |  |  |  |  |
| **Self-efficacy** |  |  |  |  |  |  |  |
| 1. I am confident that I can cope effectively with anything that comes my way |  |  |  |  |  |  |  |
| 1. I can face difficulties calmly because I trust my ability to deal with problems |  |  |  |  |  |  |  |
| 1. I can usually find several solutions when faced with a difficult problem |  |  |  |  |  |  |  |
| 1. When I'm in trouble, I can usually think of some ways to deal with it |  |  |  |  |  |  |  |
| **Conscientiousness** |  |  |  |  |  |  |  |
| 1. I am diligent in my work or study |  |  |  |  |  |  |  |
| 1. I'm a person who does things to the best of my ability |  |  |  |  |  |  |  |
| **Gratitude level** |  |  |  |  |  |  |  |
| 1. I have a lot to be thankful for in my life |  |  |  |  |  |  |  |
| 1. I have nothing to thank around the world* |  |  |  |  |  |  |  |
| 1. I appreciate all kinds of people |  |  |  |  |  |  |  |
| **Perceived organizational support** |  |  |  |  |  |  |  |
| 1. The hospital will agree to my reasonable request for a change in working conditions |  |  |  |  |  |  |  |
| 1. The hospital values my contribution to its development |  |  |  |  |  |  |  |
| 1. The hospital doesn't care what I deserve* |  |  |  |  |  |  |  |
| 1. This hospital cares more about making a profit than it does about me* |  |  |  |  |  |  |  |
| 1. The hospital tries to make my job as interesting as possible |  |  |  |  |  |  |  |
| 1. The hospital ignored any of my complaints* |  |  |  |  |  |  |  |
| 1. The hospital cannot allow me to miss work for personal reasons* |  |  |  |  |  |  |  |
| 1. The hospital did look out for my welfare |  |  |  |  |  |  |  |
| 1. The hospital pays little attention to me* |  |  |  |  |  |  |  |
| 1. The hospital didn't care how much effort I put into my work* |  |  |  |  |  |  |  |
| 1. The hospital rarely offers me opportunities for promotion* |  |  |  |  |  |  |  |
| 1. The hospital will consider my views and opinions |  |  |  |  |  |  |  |

* means that questions are scored in reverse
